# Reviewed article: Nascimento, ADF; Souza, MCL; Rossoni, SL; Santos, JH; Lopes, WA; Pujals, C; Pedroso, RB. Integrative and complementary practices, hypertension and social indicators: an ecological study, Brazil, 2023. Epidemol Serv Saude. 2026:35;e20250570

**DOI:** 10.1590/S2237-96222026v35e20250570.a

**Published:** 2026-10-05

**Authors:** Fabiana Maria de Aguiar Bello Marques

**Affiliations:** 1 Secretaria de Saúde do Recife, Recife, PE, Brazil

## Peer review A

### Questionnaire

Does the manuscript contain new and significant information to justify publication? Yes 

Does the Abstract (Summary) clearly and accurately describe the content of the article? Yes 

Is the problem significant and concisely stated? Yes 

Are the methods described comprehensively? Yes 

Are the interpretations and conclusions justified by the results? Yes 

Is adequate reference made to other work in the field? Yes 

### Manuscript Structure:

Length of article is: Adequate

Number of tables is: Adequate

Number of figures is: Adequate

### Rating

Interest: Good

Quality: Good

Originality: Good

Overall: Good

Recommendation: Minor Revision 

### Comments

Prezados (as) autores(as),

Agradeço a oportunidade de revisar este manuscrito, que aborda um tema relevante e atual. A seguir, apresento observações destinadas a aprimorar a qualidade e a clareza do trabalho.

1. Título e resumo

O título é claro e informativo.

O resumo é adequado e bem estruturado, mas sugere-se inserir com maior clareza os valores numéricos relacionados às associações (correlações e ANOVA) e especificar melhor o método na seção de “Métodos”. O período do estudo não foi explicitado no resumo.

2. Introdução

A introdução contextualiza bem o problema e a relevância do estudo. Porém, faz-se necessário, um alinhamento entre a pergunta de pesquisa e objetivo do estudo.

O estudo apresenta 02 objetivos divergentes:

i) “Avaliar as taxas de atendimento de práticas integrativas e complementares e de hipertensão arterial, bem como sua correlação com indicadores socioeconômicos no Brasil” (resumo).

iii) “Avaliar as taxas de atendimento em práticas integrativas e complementares (meditação, Tai Chi, Qigong, aromaterapia e fitoterapia) e sua relação com o manejo da hipertensão arterial no Brasil, além de analisar correlações com indicadores socioeconômicos (introdução).

Sugere-se apenas clareza na hipótese de pesquisa, pois ela está subentendida, mas não explicitada.

Recomenda-se descrição e padronização dos termos utilizados como: taxa de atendimento das PICS, taxa de atendimento por Hipertensão Arterial; taxa de utilização das PICS.

3. Métodos

Sugere-se descrever com mais detalhes os critérios de seleção das PICs, sobretudo no trecho sobre o “Mapa de Evidências”.

A descrição das análises espaciais (LISA, Kernel, Moran) está incompleta. Sugiro que explique brevemente a finalidade de cada método no texto.

O uso de dados do SISAB é pertinente, mas reconhecidamente sujeito à subnotificação. Discuta mais diretamente esse viés nas limitações.

4. Resultados

Os dados estão bem apresentados, com tabelas e figuras bem construídas e legíveis.

No entanto, assegure-se de que os dados estejam de acordo com a norma e padronizados. O período do estudo não está descrito e nem a fonte, por exemplo.

Na Tabela 1, a ordem das colunas pode ser reorganizada para facilitar a leitura.

5. Discussão, conclusões e Referências.

A discussão é coerente e crítica. Os achados são relacionados à literatura e às políticas públicas. As implicações práticas são bem traçadas, especialmente no que tange à necessidade de ampliação da oferta de PICs no SUS. A conclusão está de acordo com os resultados.

A maioria está de acordo com o estilo Vancouver.

6. Outros aspectos

A escrita é clara, com linguagem objetiva e boa organização textual. Sugere-se pequena revisão para eliminar termos redundantes e melhorar fluidez. As siglas foram bem empregadas.

O uso de IA foi informado de maneira transparente e responsável.

RECOMENDAÇÃO:

Revisão com possibilidade de aceitação após ajustes.

O manuscrito é pertinente, metodologicamente aceitável, traz achados relevantes para o SUS e apresenta boa qualidade técnica e textual. Entretanto, os autores devem atender às observações metodológicas sugeridas.

